# The patient demographics, radiographic index and surgical invasiveness for mechanical failure (PRISM) model established for adult spinal deformity surgery

**DOI:** 10.1038/s41598-020-66353-7

**Published:** 2020-06-09

**Authors:** Mitsuru Yagi, Naobumi Hosogane, Nobuyuki Fujita, Eijiro Okada, Satoshi Suzuki, Osahiko Tsuji, Narihito Nagoshi, Masaya Nakamura, Morio Matsumoto, Kota Watanabe

**Affiliations:** 1 Department of Orthopedic Surgery, Keio University School of Medicine, Tokyo, Japan; 2 Department of Orthopedic Surgery, National Hospital Organization Murayama Medical Center, Tokyo, Japan; 3 Department of Orthopedic Surgery, Kyorin University School of Medicine, Tokyo, Japan; 4 Department of Orthopedic Surgery, Fujita Health University School of Medicine, Aichi, Japan

**Keywords:** Risk factors, Outcomes research

## Abstract

Mechanical failure (MF) following adult spinal deformity (ASD) surgery is a severe complication and often requires revision surgery. Predicting a patient’s risk of MF is difficult, despite several potential risk factors that have been reported. The purpose of this study was to establish risk stratification model for predicting the MF based on demographic, and radiographic data. This is a multicenter retrospective review of the risk stratification for MF and included 321 surgically treated ASD patients (55 ± 19 yr, female: 91%). The analyzed variables were recorded for at least 2 yr and included age, gender, BMI, BMD, smoking status, frailty, fusion level, revision surgery, PSO, LIF, previous surgery, spinal alignment, GAP score, Schwab-SRS type, and rod materials. Multivariate logistic regression analyses were performed to identify the independent risk factors for MF. Each risk factor was assigned a value based on its regression coefficient, and the values of all risk factors were summed to obtain the PRISM score (range 0–12). We used an 8:2 ratio to split the data into a training and a testing cohort to establish and validate the model. MF developed in 41% (n = 104) of the training subjects. Multivariate analysis revealed that BMI, BMD, PT, and frailty were independent risk factors for MF (BMI: OR 1.7 [1.0–2.9], BMD: OR 3.8 [1.9–7.7], PT: OR 2.6 [1.8–3.9], frailty: OR 1.9 [1.1–3.2]). The MF rate increased with and correlated well with the risk grade as shown by ROC curve (AUC of 0.81 [95% CI 0.76–0.86]). The discriminative ability of the score in the testing cohort was also good (AUC of 0.86 ([95% CI 0.77–0.95]). We successfully developed an MF-predicting model from individual baseline parameters. This model can predict a patient’s risk of MF and will help surgeons adjust treatment strategies to mitigate the risk of MF.

## Introduction

Mechanical failure (MF) following surgery for adult spinal deformity (ASD) is a severe postoperative complication and often requires planned and unplanned revision surgery^1–15^. Various types of symptomatic and asymptomatic MF can be developed following ASD surgery (proximal junctional kyphosis/failure [PJK/PJF], distal junctional kyphosis [DJK], rod failure [RF]) The postoperative MF rates for ASD have been reported to be as high as 50%^1–17^. Yilgor *et al*. have described that postoperative global alignment and proportion (GAP) were significantly correlated with the MF^16^. Theologis *et al*. reported that MF increased the treatment cost by more than double in ASD surgery^17^. Several previous studies have described the poor prognosis of patients who developed neurological deficits following MF^6,10–15^. Various risk factors for MF have been reported, including age, spinal misalignment requiring a large correction, osteoporosis, and the application of pedicle subtraction osteotomy (PSO)^2,4,5,10–16,18^. However, it is still difficult to prevent or minimize the risk of complications of individuals following ASD surgery.

This study aimed to establish ASD-specific risk stratification model for predicting the risk of MF using the individual’s demographic and radiographic, and surgical description data.

## Materials and Methods

This study was approved by the institutional review board at our institutions (Keio University School of Medicine Ethics Review Committee), and all subjects consented and agreed with their inclusion. We attest that the oral and written informed consents were obtained from all these patients. The all methods were performed in accordance with the relevant guidelines and regulations.

### Patient population

We retrospectively reviewed charts and radiographs for 321 consecutive patients who underwent surgery for ASD in four academic hospitals between 2009 and 2016. For this study, we used the multicentered database established in a previous study and added 86 new patients who reached a 2-year postoperative follow-up duration^19^.

### Inclusion and exclusion criteria

Subjects were at least 20 years old at the index surgery and had a spinal deformity defined by a Cobb angle ≥ 20°, a C7 sagittal vertical axis (C7SVA) ≥ 5 cm, or pelvic tilt (PT) ≥ 25°. We included patients with at least 5 fused vertebrae, segmental instrumentation and fusion from the upper-instrumented vertebra (UIV) to the LIV (lower-instrumented vertebra), and complete 2-year follow-up data. Patients were excluded if they lacked appropriate radiographs or had multi-rod constructs, posterior tethering at the UIV + 1 vertebra, or syndromic, neuromuscular, or other pathological conditions.

### Collection of radiographic, health-related quality of life (HRQOL), and other demographic data

We collected demographic and clinical data for each patient, including age, gender, body mass index (BMI), bone mineral density (BMD), smoking status, history of joint arthroplasty (hip), and spine surgery. Frailty and comorbidities were assessed using the modified frailty index (mFI) and the Charlson comorbidity index (CCI)^20–23^. We collected the following surgical data: the SRS-Schwab ASD classification, GAP score, application of three column osteotomies, lateral lumbar interbody fusion (LIF), UIV and LIV levels, number of fused vertebrae, number of cross-connectors, material of the rods, estimated blood loss, and time of surgery. Radiographic data obtained at baseline and at the 6-week and 2-year follow-up, included the following measurements: Cobb angle, C7SVA, T4-T12, lumbar lordosis (LL), sacral slope, PT, pelvic incidence (PI), T1 pelvic angle (TPA), relative pelvic version (RPV), relative lumbar lordosis (RLL), lordosis distribution index (LDI) and relative spinopelvic alignment (RSA). As a surrogate for HRQOL, we used the Scoliosis Research Society-22r questionnaire (SRS22r) results at baseline and at the 2-year follow-up.

Of 332 candidates, 321 subjects had complete demographic and radiographic data that sufficiently captured any instances of postoperative MF and were thus included in the study cohort. We split patient samples into a training and testing cohort at an 8:2 ratio. The remaining 11 candidates were lost during follow-up, including 4 candidates who died for reasons unrelated to the surgery and were excluded from the cohort.

### Inclusion of mechanical failures

According to the previous literature, we included all MFs found on the radiographs that developed within 2 years of the operation (PJK/PJF, DJK, RF, and other implant-related complications).

### Data preparation

Subjects were categorized into two groups: those who had any MF within 2 years of the operation and those who were free of MF. We investigated the relationships between patient demographics, spinal alignment, surgical factors, and the development of complications by univariate and multivariate logistic regression analysis using the data of the training cohort (age: 53 ± 19 years; follow-up: 4.4 ± 1.7 years). We created categories based on clinical importance and on the results of unpaired t-tests and Tukey’s HSD test or the Wilcoxon ranked test where appropriate, as follows: age ≤ 60 years or > 60 years; BMI < 18.5 kg/m^2^, 18.5–25 kg/m^2^ >25 kg/m^2^; BMD T-score ≤ −1.5 or > −1.5; frailty: robust (mFI: 0), prefrail (0.09–0.21), or frail (≥ 0.27); UIV T1-T6 or T9-T11; LIV L5 and above or the pelvis; Cobb angle <70° or> 70°; C7SVA < 40 mm, 40–95 mm, or> 95 mm; PT < 20°, 20°–30°, or > 30°; PI-LL < 10°, 10°–20° or, >20°.

### Analysis of risks for mechanical failure in the patient cohort

We calculated the overall summary statistics, including the means and standard deviations for continuous variables and frequencies and percentages for categorical variables. After descriptive analysis, we analyzed the associations between potential risk factors and MF by univariate comparisons. We then created a multivariate binary logistic regression model to evaluate the adjusted associations of each potential explanatory variable and to predict the likelihood of developing MF. Clinically relevant variables and variables with a univariate significance level < 0.05 were included in the multivariate logistic regression analysis.

### Building a model to predict mechanical failure

Based on the predictors obtained from multivariate analysis, we designed a simplified, risk-stratification algorithm (PRISM) to provide a score that can be used to predict the incidence of MF. We included 6 variables in our risk-stratification model. We first established values for each risk indicator by rounding the β regression coefficient obtained in the univariate analysis to the nearest whole integer (0–3). Next, the values for all applicable risk indicators were added together with age and LIV level to establish the PRISM score (0–12). We evaluated the discriminative ability of the PRISM score based on the area under the receiver operating characteristic curve (AUROC). Linear regression analysis and the Cuzick test were performed to analyze whether there is a trend between the PRISM score and the incidence of MF. The PRISM score was used to stratify patient risk into low risk (0–1), moderate risk (2–4), high risk (5–8), and very-high risk (9–12).

### Validation of PRISM for MF

The PRISM model was applied to 64 testing samples that were not used for model development. The discriminative ability of PRISM was evaluated using AUROC analysis, linear regression algorithm, and the trend test.

### Statistical analysis

Differences between the MF and MF-free groups were compared by unpaired t-test, chi-square test, Tukey’s HSD test, and Fisher’s exact test where appropriate. Potential risk variables were analyzed by univariate and multivariate logistic regression. A correlation of the distribution of the observed MF rate for each PRISM score was created with a linear regression algorithm that best fit the data points with a 95% CI. A p value less than 0.05 with a CI of 95% was considered statistically significant^24,25^. All analyses were performed using the Statistical Package for the Social Sciences (SPSS statistics version 26.0, SPSS modeler version 18, IBM Corp., Armonk, NY).

## Results

### Patient characteristics

Among 257 training samples, MF developed in 40.5% (n = 104) of the patient cohort. The most common MF was PJK (n = 55, 21%), and the second common was RF (n = 33, 13%). Thirty-nine (38%) patients developed more than two MFs and 33 of the MF group patients (32%) required unplanned additional surgeries to treat the MFs.

### Clinical and radiographic outcomes in the mechanical failure and mechanical failure-free groups

Patients who developed MFs experienced significant improvements in HRQOL, as measured by SRS22r at the 2-year follow-up (Supplemental Table 1). However, the 2-year SRS22 scores were worse in the MF group than in the MF-free group except for the mental-health subdomain (Supplemental Table 1).

### Risk analysis for mechanical failure

Comparisons of the demographic and radiographic data between the MF and MF-free groups indicated different distributions of age, BMI, BMD, frailty, CCI, baseline and 2-year postoperative sagittal spinal alignment, curve type, and surgical details (Table 1 and Supplemental Table 2). Univariate analyses revealed the following significant risk factors for the development of MF, presented here in order of the odds ratio (OR): Curve type N, type L, LIV, PT, age, frailty, PI-LL, BMD, PSO, UIV, C7SVA, and LIF (Table 2).

**Table 1:** Comparisons of demographic data and surgical descriptions between the mechanical failure-free and mechanical failure groups in the training cohort.

	MF-free	MF	Total	P value
Age	46.6 ± 19.6 [20, 78]	62.9 ± 12.7 [23, 78]	53.2 ± 18.9 [20, 78]	<0.01*
BMI (kg/m^**2**^)	20.2 ± 2.9 [15.2, 28.9]	22.7 ± 4.0 [11.9, 31.6]	21.2 ± 3.6 [11.9, 31.6]	<0.01*
BMD (T-score)	−0.6 ± 1.0 [−3.0, 2.4]	−1.2 ± 0.9 [−3.4, 1.2]	−0.8 ± 1.0 [−3.4, 2.4]	<0.01*
mFI	0.04 ± 0.07 [0, 0.36]	0.11 ± 0.13 [0, 0.64]	0.07 ± 0.11 [0, 0.64]	<0.01*
CCI	1.0 ± 1.3 [0, 8]	1.9 ± 1.7 [0, 9]	1.4 ± 1.6 [0, 9]	<0.01*
TOS (min.)	245 ± 84 [89, 495]	282 ± 923 [99, 542]	248 ± 92 [89, 542]	<0.01*
EBL (mL)	557 ± 379 [80, 2500]	770 ± 551 [150, 3000]	643 ± 467 [80, 3000]	<0.01*
Level fused	9.1 ± 2.9 [5, 16]	10.0 ± 2.7 [5, 18]	9.5 ± 2.8 [5, 16]	0.01*
**Baseline**				
C7SVA (*mm*)	36.8 ± 57.0	85.3 ± 65.6	56.6 ± 65.1	<0.01*
PT (*°*)	22.6 ± 11.5	32.9 ± 22.1	26.9 ± 12.8	<0.01*
PI-LL (*°*)	20.2 ± 20.8	39.1 ± 23.7	27.7 ± 23.8	<0.01*
**Immediately postop**				
C7SVA (*mm*)	7.9 ± 33.1	23.6 ± 35.6	14.3 ± 34.9	<0.01*
PT (*°*)	18.9 ± 7.8	21.6 ± 8.7	20.0 ± 8.3	0.01*
PI-LL (*°*)	9.2 ± 11.1	11.8 ± 16.7	10.2 ± 13.6	0.15
PJA (*°*)	5.3 ± 5.3	6.6 ± 6.9	6.0 ± 6.3	0.18
RPV (*°*)	−9.1 ± 9.2	−12.4 ± 8.1	−10.5 ± 9.9	<0.01*
RLL (*°*)	−19.7 ± 10.8	−21.8 ± 15.6	−20.6 ± 13.0	0.20
LDI (*°*)	63.5 ± 53.8	69.0 ± 57.7	65.7 ± 55.4	0.43
RSA (*°*)	−2.6 ± 8.8	4.2 ± 10.0	0.2 ± 9.9	<0.01*
GAP score	5.2 ± 2.7	5.9 ± 3.3	5.5 ± 3.0	0.07
**2-year postop**				
C7SVA (*mm*)	12.5 ± 40.5	65.3 ± 56.3	34.1 ± 54.2	<0.01*
PT (*°*)	19.4 ± 8.0	26.6 ± 11.0	22.4 ± 9.9	<0.01*
PI-LL (*°*)	9.2 ± 13.0	14.6 ± 16.1	11.4 ± 14.5	<0.01*
PJA (*°*)	7.4 ± 6.4	15.0 ± 11.7	11.5 ± 10.3	<0.01*

Mean and standard deviations. Range in brackets. P values indicate comparisons of the values between the MF-free and MF groups. *statistically significant.

**Table 2:** Univariate logistic regression analysis for the risk of mechanical failure following ASD surgery.

	Regression coefficient	P value	OR
**Age**			
<60 yrs		Reference	
≧60 yrs	1.33 (0.27)	<0.01*	3.79 [2.23, 6.44]
**BMI** ***(p*** **for trend)**	0.93 (0.23)	<0.01*	2.54 [1.63, 3.98]
Lean		Reference	
Moderate	0.95 (0.35)	<0.01*	2.59 [1.30, 5.18]
Overweight	1.87 (0.46)	<0.01*	6.46 [2.63, 15.84]
**BMD**			
T-score > −1.5		Reference	
T-score ≦−1.5	1.51 (0.23)	<0.01*	4.50 [2.44, 8.31]
**Frailty (*p*** **for trend)**	1.29 (0.24)	<0.01*	3.62 [2.26, 5.81]
Robust		Reference	
Prefrail	1.49 (0.30)	<0.01*	4.44 [2.47, 8.00]
Frail	2.08 (0.60)	<0.01*	8.00 [2.45, 26.10]
**Prior spine surgery**			
Primary		Reference	
Revision	0.13 (0.52)	0.80	1.14 [0.41, 3.15]
**Prior hip replacement**			
No THA		Reference	
THA	1.13 (0.63)	0.07	3.14 [0.91, 10.59]
**UIV**		Reference	
Above T8			
Below T9	0.94 (0.27)	<0.01*	2.57 [1.51, 4.36]
**LIV**			
Above L5			
Pelvis	1.69 (0.28)	<0.01*	5.39 [3.12, 9.31]
**Type of distal screw**			
Iliac screw		Reference	
S2AI	−0.19 (0.41)	0.64	0.82 [0.37, 1,85]
**LIF**	0.76 (0.35)	0.03*	2.15 [1.08, 4.28]
**PSO**	1.07 (0.44)	0.01*	2.91 [1.23, 6.87]
**Rod materials**			
Ti alloy	—	Reference	
CoCr	−0.20 (0.26)	0.44	0.82 [.50, 1.35]
**No. of cross-connectors**			
0		Reference	
1	−0.14 (0.39)	0.72	0.87 [.40, 1.87]
2	−0.51 (0.40)	0.21	0.60 [.27, 1.32]
**SRS-Schwab type**			
Type T		Reference	
Type D	1.55 (0.49)	<0.01*	4.71 [1.80, 12.29]
Type L	1.75 (0.50)	<0.01*	5.77 [2.17, 15.31]
Type N	2.71 (0.52)	<0.01*	15.00 [5.39, 41.71]
**Sagittal modifier**			
**PI-LL (*p*** **for trend)**	0.95 (0.18)	<0.01*	3.37 [2.35, 4.84]
PI-LL (0)		Reference	
PI-LL (+)	0.75 (0.48)	0.12	2.12 [0.83, 5.41]
PI-LL (++)	1.86 (0.36)	<0.01*	6.43 [3.19, 12.97]
**PT (*p*** **for trend)**	1.33 (0.27)	<0.01*	3.79 [2.23, 6.44]
PT (0)		Reference	
PT (+)	1.53 (0.40)	<0.01*	4.61 [2.11, 10.10]
PT (++)	2.51 (0.39)	<0.01*	12.30 [5.74, 26.35]
**SVA (*p*** **for trend)**	0.90 (0.17)	<0.01*	2.45 [1.76, 3.41]
SVA (0)		Reference	
SVA (+)	1.35 (0.32)	<0.01*	3.86 [2.05, 7.27]
SVA (++)	1.74 (0.34)	<0.01*	5.70 [2.92, 11.11]
**GAP score (*p*** **for trend)**	0.15 (0.17)	0.39	1.16 [0.83, 1.64]
PR		Reference	
MD	−0.48 (0.35)	0.17	0.62 [0.31, 1.23]
SD	0.14 (0.35)	0.69	1.15 [0.58, 2.38]

OR: Odds ratio. Standard error in parentheses. 95% confidence interval in brackets. *statistically significant. PR: proportioned. MD: moderately disproportioned. SD: severely disproportioned. Ti alloy: titanium alloy. CoCr: cobalt-chrome. THA: total hip replacement.

Among these risk factors, the multivariate analysis identified that BMD, PT, frailty, and BMI as independent risk factors for MF (BMD: OR 3.8 [1.9–7.7], PT: OR 2.6 [1.8–3.9], frailty: OR 1.9 [1.1–3.2], BMI: OR 1.7 [1.0–2.9], Table 3).

**Table 3:** Multivariate logistic regression analysis for the risk of mechanical failure following ASD surgery.

	Regression coefficient	P value	OR
BMI	0.54 (0.27)	0.04*	1.72 [1.02, 2.92]
BMD (T score < −1.5)	1.33 (0.36)	<0.01*	3.79 [1.88, 7.66]
SRS-Schwab modifier: PT	0.97 (0.21)	<0.01*	2.63 [1.76, 3.93]
Frailty	0.64 (0.27)	0.02*	1.89 [1.12, 3.21]

OR: Odds ratio. Standard error in parentheses. 95% confidence interval in brackets. *statistically significant.

### Building and validating a model to predict mechanical failure

**Figure 1:**
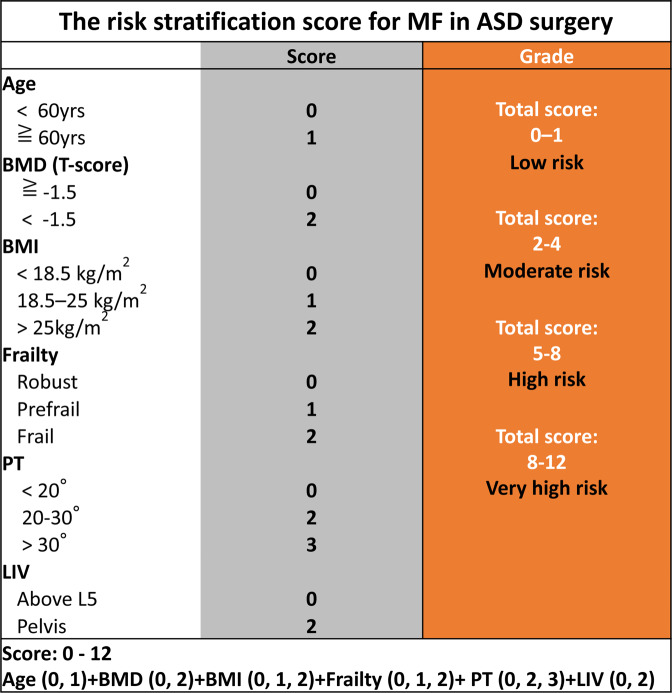
Risk-grading system for mechanical failure following ASD surgery. The risk stratification score was used to stratify the risk into low risk (risk score 0–1), moderate risk (risk score 2–4), high risk (risk score 5–8), and very high risk (risk score 9–12).

**Figure 2:**
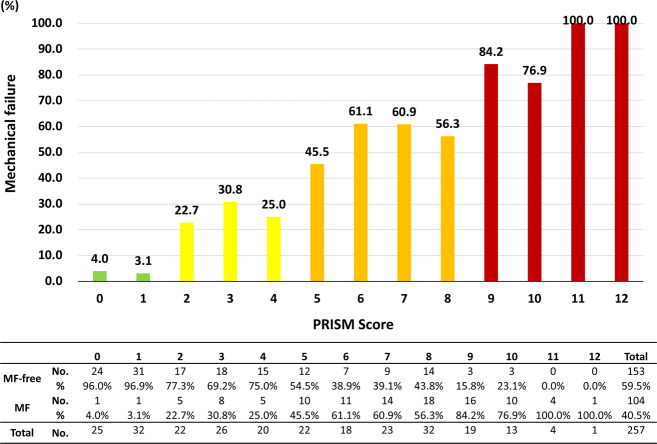
The distribution of mechanical failure in the training cohort, stratified by score relative to the observed mechanical failure rate. The mechanical failure rate increased with the score. A statistically significant trend between the mechanical failure rate and the score was observed (*p* for trend ≤ 0.001, Cuzick test).

We created a surgical risk grading system with 6 risk variables, including 4 variables identified in the multivariate analysis, namely, BMI, BMD, baseline PT, and frailty, and 2 clinically important variables, patient age and level of LIV (pelvis). The PRISM score was determined as the sum of the values of the risk variables (Fig. 1). Thirty-seven percent of the patients were classified as grade high risk, 27% as moderate risk, 22% as low risk, and 14% as very high risk (Fig. 2). MF increased exponentially as the PRISM score worsened, and the linear regression algorithm that best fit the data points with a 95% CI confirmed an excellent correlation between the PRISM score and the actual development of MF, using the following regression model: y = 1.66 + 8.29x and r^2^ = 0.956.

**Figure 3:**
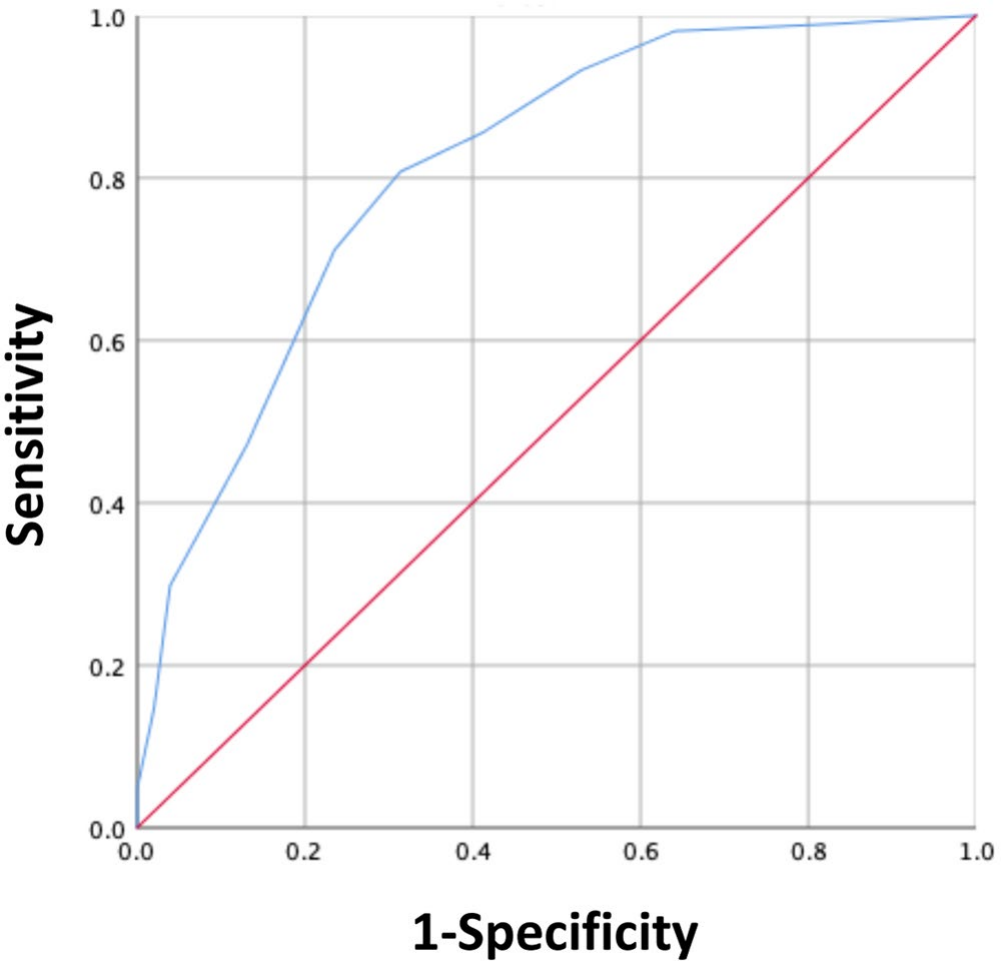
The distribution of the score and receiver operating characteristic (ROC) analysis in the training cohort relative to the observed mechanical failure rate for each score. ROC curve of the mechanical failure rate for the score (red line) in the training cohort. The area under the ROC curve (AUROC) was 0.812, stander error = 0.026, *p* ≤ 0.001, 95% CI = 0.763-0.864 for the score.

**Figure 4:**
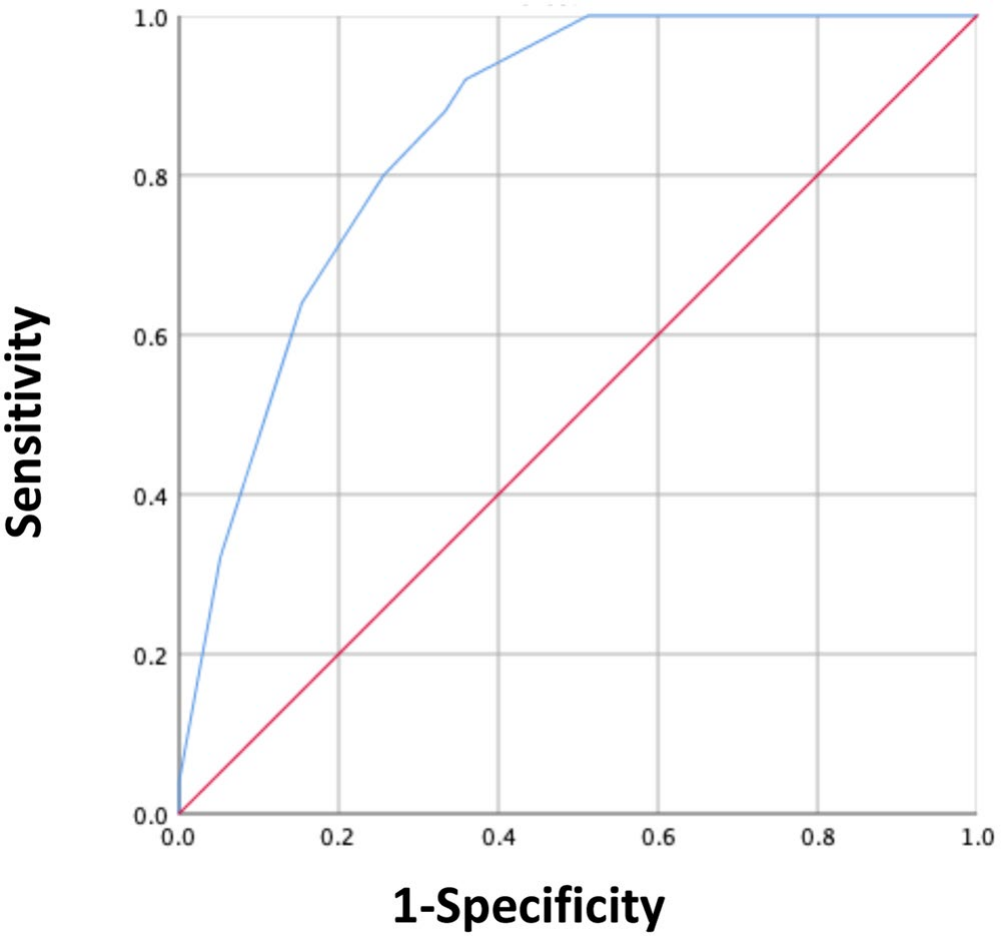
The distribution of scores and receiver operating characteristic (ROC) analysis in the testing cohort relative to the observed mechanical failure rate for each score. ROC curve of s mechanical failure s for the scores (red line) in the testing samples. The area under the ROC curve (AUROC) was 0.855, stander error = 0.046, *p* ≦0.001, 95% CI = 0.765-0.945 for the score.

**Figure 5:**
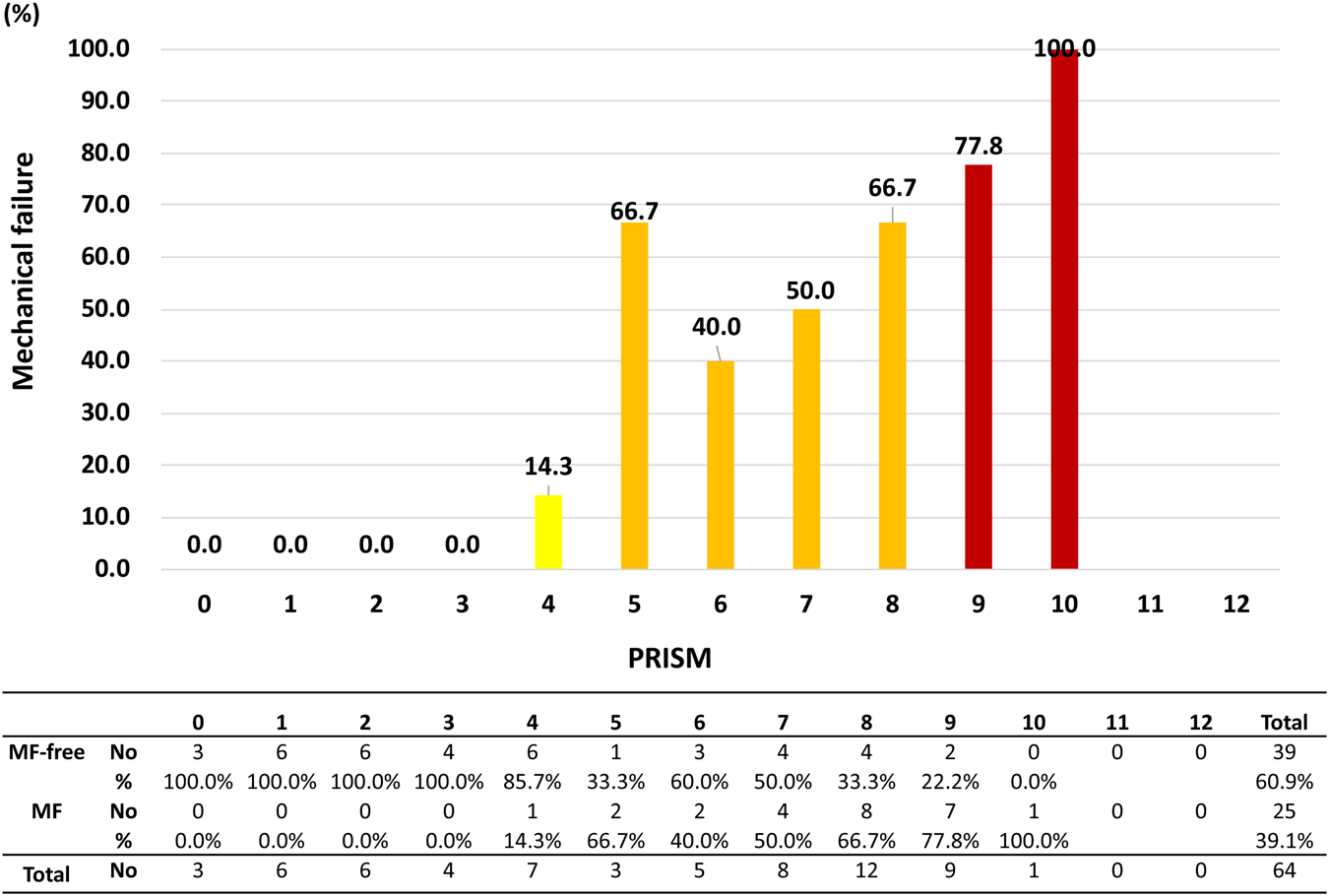
Distribution of scores and grades in the testing cohort relative to the observed mechanical failure rate. The incidence of mechanical failure increased with the score. A statistically significant trend between the mechanical failure rate and the score was observed (*p* for trend ≤ 0.001, Cuzick test).

The PRISM score also showed excellent accuracy for predicting the incidence of MF, with an AUROC of 0.812 (95% CI 763-0.864, Fig. 3 and Supplemental Table 3). In addition, the trend analysis showed excellent correlation with the incidence of MF and PRISM score (Cuzick test, P < 0.001). Internal validation with the testing sample for PRISM showed good model fit for the prediction of MF with excellent discriminating ability (y = 13.9 + 10.5x and r^2^ = 0.866, AUROC 0.855 [95% CI 765-0.945], Figs 4 and 5 and Supplemental Table 4).

## Discussion

Mechanical failure is the most common surgical complication after ASD surgery^1–15^. In this study, MF developed in 40.5% of patients following ASD surgery. Among them, 39% of patients in the MF group developed multiple MFs during follow-up, and 30% of patients in the MF group required unplanned reoperation. Crawford *et al*. described that 24% of patients required an unplanned reoperation following ASD surgery, and the most common indication for reoperation was RF^1^. Inoue *et al*. described that the most frequent type of MF associated with reoperation was PJF^25^. In the present study, approximately 36% of RFs developed 3 years or longer after surgery, and 70% of them were not associated with either significant alteration in HRQOL or progressive deformity. Following the previous literature, the clinical outcomes of patients who developed MF were inferior to those of MF-free patients at 2 years after surgery. Soroceanu *et al*.^26^ described that MF significantly affected HRQOLs in 245 consecutive ASD surgeries. Lertudomphonwanit also described that the MF group had less overall improvement in HRQOLs than did the MF free group^2^. Recently, several cost-utility analyses of the surgical treatment for ASD have been performed^17,27,28^. Yagi *et al*. described that revision surgery for ASD increased the 2-year total cost by approximately 30% and significantly decreased the cost-effectiveness of the surgery^27^. Safaee *et al*. also described the significant alteration in cost-effectiveness of ASD surgery for those who required revision surgery^28^. Taken together, to predict the risk of MF and to reduce this common but significant surgical complication, it is essential to mitigate the risk of surgical complications and to improve the outcome of surgical treatment of ASD surgery.

Several potential risk factors for developing MF have been reported and confirmed in the previous literature^2,4,5,10–16,18^. One can argue that MF includes various implant-related complications, and therefore, the risk analysis should be oriented to each type of MF. However, there is an overlap among the risk of each type of MF. Yagi *et al*. described BMD, age, a large amount of sagittal alignment correction, and pelvic fusion as independent risk factors for PJF^13–15^. On the other hand, Smith *et al*. described these factors as independent risk factors for RF. In the present study, 38% of the MF group patients actually developed 2 or more types of MFs during follow-up^5,29,30^. This higher incidence of the development of multiple MFs in the same patient observed in the present study strongly supports the presence of common risk factors among the various types of MFs.

In the present study, we established a mechanical failure predictive model based on individual demographics, baseline spinal alignment, and surgical descriptive data. The advantages of the model include the feasibility of the scoring system. This scoring system consists only of preoperative values and therefore can be fully assessed before surgery. The model also showed good accuracy for predicting the incidence of MF in both the training and testing cohorts, with an AUC of 0.81 in the training subjects and of 0.86 in the testing subjects.

This study was limited by its retrospective design and the lack of external validation, which precludes drawing firm conclusions about our model’s predictive power for MF. However, we enrolled consecutive patients from a prospective database and analyzed the patients retrospectively, which is the most common method for investigating how a factor affects outcomes and complications in clinical research when randomized controlled trials are not possible. Moreover, MF is a radiographic complication and can therefore be retrospectively corrected from the periodical radiographs. In the present study, we enrolled patients from 7 surgeons in 3 academic hospitals in an East Asian country, so the patients were mostly Asian. Therefore, our results cannot necessarily be extrapolated to all other hospital settings. It is widely accepted that BMD is different between races^31^. Therefore, either adjustment of BMD index or addition of race index may be necessary to further improve the accuracy of PRISM in the other different populations. Further analyses including different patient populations are necessary to validate the predictive power of our model for MF following ASD surgery. Despite these limitations, the present study clearly showed the good predictive probability of this newly created model, which is based on demographic, radiographic, and surgical description data, for predicting MF following ASD surgery.

## Conclusion

The newly established risk-stratification scoring model can predicted MF following ASD surgery using individual demographics and radiographic parameters as well as surgical description data that would normally be collected routinely when considering surgical treatment for a patient with ASD. This model can help surgeons identify patients with a high risk of MF and treat modifiable risk variables to mitigate the risk of MF following ASD surgery.

## Supplementary information

SUPPLEMENTAry Information.
