# Supplementary material for: The patient demographics, radiographic index and surgical invasiveness for mechanical failure (PRISM) model established for adult spinal deformity surgery
Source: Sci Rep. 2020 Jun 9;10:9341. doi: 10.1038/s41598-020-66353-7 (PMC7283344; doi:10.1038/s41598-020-66353-7)
Supplement: Supplementary file 1 — SUPPLEMENTAry Information. [file 41598_2020_66353_MOESM1_ESM.docx]

**The patient demographics, radiographic index and surgical invasiveness for mechanical failure (PRISM) model established for adult spinal deformity surgery**

*Authors:*

Mitsuru Yagi MD, PhD^1,2^, Naobumi Hosogane MD, PhD^3^, Nobuyuki Fujita MD, PhD^4^, Eijiro Okada M.D., Ph.D.^1^, Satoshi Suzuki M.D., Ph.D.^1^, Osahiko Tsuji M.D., Ph.D.^1^, Narihito Nagoshi M.D., Ph.D.^1^, Masaya Nakamura MD, PhD^1^, Morio Matsumoto MD, PhD^1^, Kota Watanabe MD, PhD^1^

*Affiliations:*

^1^ Department of Orthopedic Surgery, Keio University School of Medicine, Tokyo JAPAN

^2^ Department of Orthopedic Surgery, National Hospital Organization Murayama Medical Center, Tokyo JAPAN

^3^ Department of Orthopedic Surgery, Kyorin University School of Medicine, Tokyo JAPAN

^4^ Department of Orthopedic Surgery, Fujita Health University School of Medicine, Aichi JAPAN

*Corresponding author:*

Kota Watanabe, MD, PhD

35 Shinanomachi, Shinjyuku, Tokyo, Japan

Tel: +81.3.3353.1221; fax: +81.3.3353.1232; e-mail: kw197251@keio.jp

**SUPPLEMENTAL TABLES**

Supplemental Table 1. Comparisons of the baseline and 2-year postoperative ASDs with or without mechanical failure in the training cohort.

|  | **MF-free** | **MF** | **Total** | **P value** |
| --- | --- | --- | --- | --- |
| **Baseline** |  |  |  |  |
| SRS22 function | 3.48 ± .95 | 2.85 ± .74 | 3.25 ± .93 | <.01* |
| SRS22 pain | 3.26 ± .94 | 2.81 ± .83 | 3.09 ± .93 | <.01* |
| SRS22 self-image | 2.40 ± .67 | 2.28 ± .71 | 2.36 ± .69 | .24 |
| SRS22 mental health | 3.06 ± 1.00 | 2.75 ± .82 | 2.95 ± .95 | .02* |
| SRS22 satisfaction | 3.42 ± .77 | 3.47 ± .83 | 3.44 ± .79 | .68 |
| SRS22 total | 3.11 ± .67 | 2.72 ± .55 | 2.97 ± .65 | <.01* |
| **2-year postop** |  |  |  |  |
| SRS22 function | 3.98 ± .75 | 3.57 ± .68 | 3.81 ± .75 | <.01* |
| SRS22 pain | 3.96 ± .73 | 3.63 ± .85 | 3.82 ± .80 | <.01* |
| SRS22 self-image | 3.88 ± .64 | 3.50 ± .84 | 3.72 ± .75 | <.01* |
| SRS22 mental health | 3.89 ± .77 | 3.73 ± .82 | 3.83 ± .79 | .14 |
| SRS22 satisfaction | 4.06 ± .75 | 3.85 ± .86 | 3.97 ± .80 | .05* |
| SRS22 total | 3.94 ± .61 | 3.63 ± .67 | 3.81 ± .65 | <.01* |
| Mean and standard deviations. P values indicate comparisons of the values between the MF-free and MF groups. *statistically significant. | | | | |

Supplemental Table 2. Comparisons of patient demographic data and surgical invasiveness in the training samples.

|  | | **MF-free** | **MF** | **Total** | **P value** |
| --- | --- | --- | --- | --- | --- |
| **Sex** | Female | 139 (59%) | 97 (41%) | 236 (92%) | .64 |
|  | Male | 14 (67%) | 7 (33%) | 21 (8%) |  |
| **Smoker** | No | 147 (59%) | 102 (40%) | 249 (97%) | .37 |
|  | Yes | 6 (75%) | 2 (25%) | 8 (3%) |  |
| **History of spine surgery** | Primary | 146 (61%) | 95 (39%) | 241 (94%) | .29 |
|  | Revision | 7 (47%) | 8%53%) | 15 (6%) |  |
| **History of hip arthroplasty** | No | 149 (61%) | 96 (39%) | 245 (95%) | .07 |
|  | Yes | 4 (33%) | 8 (66%) | 12 (5%) |  |
| **UIV** | Above T8 | 78 (72%) | 30 (28%) | 108 (42%) | <.01* |
|  | Below T9 | 75 (50%) | 74 (50%) | 149 (58%) |  |
| **LIV** | Above L5 | 118 (75%) | 40 (25%) | 158 (61%) | <.01* |
|  | Pelvis | 35 (35%) | 64 (65%) | 99 (39%) |  |
| **Type of distal screw** | Iliac screw | 32 (32%) | 63 (67%) | 95 (37%) | .09 |
|  | S2AI | 3 (75%) | 1 (25%) | 4 (2%) |  |
| **LIF** | No | 136 (62%) | 82 (38%) | 218 (85%) | .02* |
|  | Yes | 17 (44%) | 22 (56%) | 39 (15%) |  |
| **PSO** | No | 144 (62%) | 88 (38%) | 232 (90%) | .01* |
|  | Yes | 9 (36%) | 16 (64%) | 25 (10%) |  |
| **Rod materials** | Ti alloy | 66 (57%) | 50 (43%) | 116 (45%) | .45 |
|  | CoCr | 87 (62%) | 54 (38%) | 141 (55%) |  |
| **No. of cross-connectors** | 0 | 18 (53%) | 16 (47%) | 34 (13%) | .79 |
|  | 1 | 66 (56%) | 51 (44%) | 117 (46%) |  |
|  | 2 | 69 (65%) | 37 (35%) | 106 (41%) |  |
| **SRS-Schwab type** | Type T | 45 (88%) | 6 (12%) | 51 (20%) | <.01* |
|  | Type D | 51 (61%) | 32 (39%) | 83 (32%) |  |
|  | Type L | 39 (57%) | 30 (44%) | 69 (27%) |  |
|  | Type N | 18 (33%) | 36 (67%) | 54 (21%) |  |
| **Sagittal modifier** |  |  |  |  |  |
| Modifier: PI-LL | 0 | 60 (82%) | 13 (18%) | 73 (28%) | <.01* |
|  | + | 27 71%) | 11 (29%) | 38 (15%) |  |
|  | ++ | 64 (44%) | 82 (56%) | 146 (57%) |  |
| Modifier: PT | 0 | 73 (87%) | 11 (13%) | 84 (33%) | <.01* |
|  | + | 46 (60%) | 31 (40%) | 77 (30%) |  |
|  | ++ | 33 (35%) | 63 (66%) | 96 (37%) |  |
| Modifier: SVA | 0 | 90 (78%) | 25 (22%) | 115 (45%) | <.01* |
|  | + | 37 (49%) | 39 (51%) | 76 (30%) |  |
|  | ++ | 25 (38%) | 41 (62%) | 66 (26%) |  |
| **GAP score** | PR | 28 (56%) | 22 (44%) | 50 (19%) | .08 |
|  | MD | 74 (67%) | 36 (33%) | 110 (43%) |  |
|  | SD | 51 (53%) | 46 (47%) | 97 (38%) |  |
| Percentage in parentheses. P values indicate comparisons of the values between the MF-free and MF groups. *statistically significant. PR: proportioned. MD: moderately disproportioned. SD: severely disproportioned. S2AI: S2 ala-iliac screw. Ti alloy: titanium alloy. CoCr: cobalt-chrome. THA: total hip replacement. | | | | | |

Supplemental Table 3. Cutoff values of the ROC curve along with specificity and 1-sensitivity of risk stratification scores for mechanical failure in the training cohort.

| **Cutoff value** | **Sensitivity** | **1-Specificity** |
| --- | --- | --- |
| -1.00 | 1.000 | 1.000 |
| 0.50 | 0.990 | 0.843 |
| 1.50 | 0.981 | 0.641 |
| 2.50 | 0.933 | 0.529 |
| 3.50 | 0.856 | 0.412 |
| 4.50 | 0.808 | 0.314 |
| 5.50 | 0.712 | 0.235 |
| 6.50 | 0.606 | 0.190 |
| 7.50 | 0.471 | 0.131 |
| 8.50 | 0.298 | 0.039 |
| 9.50 | 0.144 | 0.020 |
| 10.50 | 0.048 | 0.000 |
| 11.50 | 0.010 | 0.000 |
| 13.00 | 0.000 | 0.000 |

**Supplemental Table 4. Cutoff values of the ROC curve along with specificity and 1-sensitivity of risk stratification scores for mechanical failure in the testing cohort.**

| **Cutoff value** | **Sensitivity** | **1-Specificity** |
| --- | --- | --- |
| -1.00 | 1.000 | 1.000 |
| 0.50 | 1.000 | 0.923 |
| 1.50 | 1.000 | 0.769 |
| 2.50 | 1.000 | 0.615 |
| 3.50 | 1.000 | 0.513 |
| 4.50 | 0.920 | 0.359 |
| 5.50 | 0.880 | 0.333 |
| 6.50 | 0.800 | 0.256 |
| 7.50 | 0.640 | 0.154 |
| 8.50 | 0.320 | 0.051 |
| 9.50 | 0.040 | 0.000 |
| 11.00 | 0.000 | 0.000 |
| -1.00 | 1.000 | 1.000 |
| 0.50 | 1.000 | 0.923 |
